# A high-resolution flux-matrix model describes the spread of diseases in a spatial network and the effect of mitigation strategies

**DOI:** 10.1038/s41598-022-19931-w

**Published:** 2022-09-24

**Authors:** Guillaume Le Treut, Greg Huber, Mason Kamb, Kyle Kawagoe, Aaron McGeever, Jonathan Miller, Reuven Pnini, Boris Veytsman, David Yllanes

**Affiliations:** 1 Chan Zuckerberg Biohub, 499 Illinois Street, San Francisco, CA 94158 USA; 2 Department of Physics, Kadanoff Center for Theoretical Physics, University of Chicago, Chicago, IL 60637 USA; 3 Okinawa Institute of Science and Technology, Onna-son, Okinawa 904-0495 Japan; 4 Chan Zuckerberg Initiative, Redwood City, CA 94063 USA; 5 School of Systems Biology, George Mason University, Fairfax, VA 22030 USA; 6 Instituto de Biocomputación y Física de Sistemas Complejos (BIFI), 50018 Zaragoza, Spain

**Keywords:** Population dynamics, Biological physics, Applied mathematics

## Abstract

Propagation of an epidemic across a spatial network of communities is described by a variant of the SIR model accompanied by an intercommunity infectivity matrix. This matrix is estimated from fluxes between communities, obtained from cell-phone tracking data recorded in the USA between March 2020 and February 2021. We apply this model to the SARS-CoV-2 pandemic by fitting just one global parameter representing the frequency of interaction between individuals. We find that the predicted infections agree reasonably well with the reported cases. We clearly see the effect of “shelter-in-place” policies introduced at the onset of the pandemic. Interestingly, a model with uniform transmission rates produces similar results, suggesting that the epidemic transmission was deeply influenced by air travel. We then study the effect of alternative mitigation policies, in particular restricting long-range travel. We find that this policy is successful in decreasing the epidemic size and slowing down the spread, but less effective than the shelter-in-place policy. This policy can result in a pulled wave of infections. We express its velocity and characterize the shape of the traveling front as a function of the epidemiological parameters. Finally, we discuss a policy of selectively constraining travel based on an edge-betweenness criterion.

## Introduction

When plague hit Florence in August 1630, the Florentine authorities made a number of high-stakes decisions which proved highly effective^1^. One of the reasons the Florence *Sanitá* could organize this response was the ample time they had, forewarned as they were by the Milanese authorities in November 1629. Today’s public-health authorities work under much more compressed timescales, as evidenced by the SARS-CoV-2 pandemic. Long-distance travel radically changes the dynamics of spreading, which raises a number of questions about the spatial dynamics of transmission in modern times. Epidemic outbreaks in the last two decades have provided the scientific community with a wealth of material to study these questions, going beyond the classic Susceptible-Infected-Recovered (SIR) theory with perfect mixing^2–6^. Several studies have shown how the total epidemic size can be affected by factors such as inhomogeneity in transmission rates^7–14^ or in the mode of transmission^15,16^. Classically, motion of individuals was taken into account by introducing diffusion terms in the standard SIR equations, allowing the emergence of spatio-temporal patterns^17–20^. Recently, in the context of the SARS-CoV-2 pandemic, such approaches have been especially valuable in order to study the effect of containment policies such as lockdown and quarantine^21,22^. These models are, however, limited in that they do not, in principle, take long-distance air travel into account. Several works have, therefore, considered disease spread in a network, typically constructed from air-traffic data^19,23,24^, where edges can connect locations separated by large geographical distances. This approach can lead to very accurate predictions at the country scale^25^ but predictions at finer scales remain challenging. Another study considering human mobility emphasized how spatial variation in public-health infrastructure reflected on epidemiological parameters can affect the dynamics of spread to different countries^26^. Data-based studies of epidemic spread and the impact of social distancing through the analysis of social-network structure have also been very informative^16,27–29^.

A recent paper by Chang et al.^30^ obtained a model for the spatio-temporal spread of a disease at a high spatial resolution by using extensive mobile tracking information to identify physical interactions between individuals. Chang et al. showed that the actual spread of the SARS-CoV-2 epidemic can be well explained from the mobility data of individuals. The model relied on the simulation of interactions among individuals on a bipartite graph where nodes, representing locations at a very fine spatial resolution, are divided in two sets: Census Block Groups (residential areas) and Points Of Interest (non-residential), each of them having its own transmission rate. The model was fitted to reproduce known reported cases of COVID-19 in 10 metropolitan areas, and could then be used to make short-term predictions about the spreading or study the effect of different mitigation strategies.

Here we take an approach similar to that of Chang et al., using mobility data to calibrate a model for disease spread. However, we investigate this propagation at the scale of a large country, the USA, rather than metropolitan areas. Specifically, we introduce a spatial SIR epidemiological model in which effective transmission rates between $N={2^{10}}$ communities are computed from mobility data of individuals belonging to these communities. We show that this model captures well the spread of the SARS-CoV-2 epidemic. Remarkably, we find that a simple model consisting of an interaction frequency dropping under the effect of lockdown, and of a single flux matrix encoding the travel of individuals, faithfully reproduces the reported cases of COVID-19 both globally and locally in each community. Strikingly, the SARS-CoV-2 epidemic spreads in a delocalized fashion, infecting distant communities very quickly. Moreover, an even simpler model with uniform transmission rates between the communities gives results very close to the model based on mobility data, emphasizing the prevalent role air travel played in the spread of the SARS-CoV-2 epidemic. We then study how interventions that change travel patterns can localize epidemics. In particular, we investigate the hypothetical effect of a policy preventing long-distance travel. In addition to “flattening” the curve, spreading through nearest-neighbor interactions creates traveling waves, which we characterize both analytically and numerically. These results allow us to discuss which interventions are more effective, limiting short-range contacts (a lockdown), or limiting long-range trips (a quarantine). We also propose an alternative mitigation strategy based on an edge-betweenness criterion.

## Model

**Figure 1:**
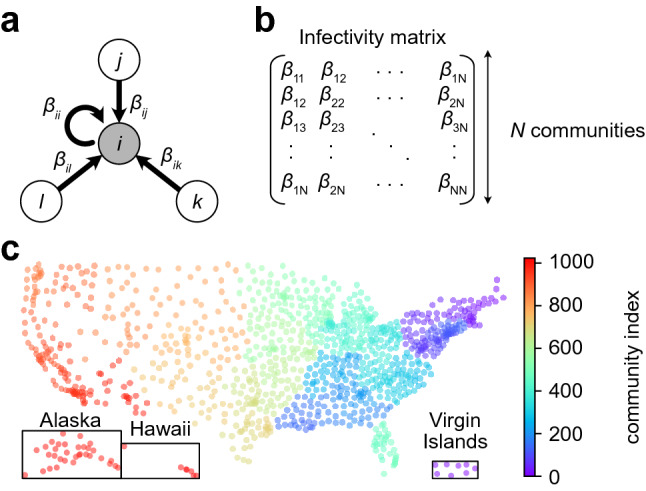
Model for the spreading of SARS-CoV-2 in a network of communities in the USA. (**a**) One community *i* interacting with three other communities *j*, *k* and *l*, with the transmission rates $\beta _{ij}$, $\beta _{ik}$ and $\beta _{il}$ respectively. (**b**) Infectivity matrix. (**c**) $N=2^{10}$ communities in the USA. Each community aggregates a number of Census Block Groups (CBGs).

We consider a metapopulation model of *N* communities numbered 1, 2,..., *N*. Denoting by $S_a$, $I_a$ and $R_a$ the numbers of susceptible, infected and recovered individuals in community *a*, the standard SIR equations read:

1 $$\begin{aligned} \frac{\mathrm {d}S_a}{\mathrm {d}t} = - S_a \sum \limits _b \beta _{ab} \frac{I_b}{M_b},\quad \frac{\mathrm {d}R_a}{\mathrm {d}t} = \gamma I_a,\quad \frac{\mathrm {d}I_a}{\mathrm {d}t} = - \frac{\mathrm {d}S_a}{\mathrm {d}t} - \frac{\mathrm {d}R_a}{\mathrm {d}t}, \end{aligned}$$

where $\beta _{ab}$ is the transmission rate from infected individuals in community *b* to susceptible individuals in community *a* and $\gamma$ is the recovery rate, assumed to be the same for all communities. Diagonal elements of the infectivity matrix $[\beta _{ab}]$ describe intracommunity infections, while off-diagonal elements describe inter-community infections (Fig. 1a,b). We also introduce the local epidemic sizes $T_a = I_a + R_a$. The total population in each community $M_a$ is constant through time,

2 $$\begin{aligned} S_a(t) + I_a(t) + R_a(t) = M_a. \end{aligned}$$

The model in Eq. (1) has been extensively studied^31–35^. We show in the Supplementary Information (SI) that the dynamics can be reduced to an ODE of just one *N*-vector variable, and the endemic equilibrium can be obtained by solving a transcendental equation involving the infectivity matrix $[\beta _{ab}]$.

In order to estimate the infectivity matrix, we used mobility data compiled by SafeGraph^36^, tracking the location of about 20 million USA cell phones between March 2020 and February 2021. The locations consist of more than 200,000 Census Block Groups (CBGs). Each cell phone is assigned for physical residence the location where it spent the most time, and daily visits to other locations are recorded. For computational purposes, we coarse-grained the physical locations into $N = {2^{10}}$ communities, which are shown in Fig. 1c. Let $f_{ab}$ be the number of individuals from community *a* visiting community *b* per unit of time. We will assume that

3 $$\begin{aligned} f_{ab}\ll M_a, \quad f_{ab} \ll M_b, \quad \text { for all $a$ and $b$}. \end{aligned}$$

The variation in susceptible individuals in community *a* due to new infections during the time interval $\Delta t$ has the form:

4 $$\begin{aligned} S_a(t + \Delta t) - S_a(t) = -S_a(t) \times \mathrm {Pr}\left( \text {meeting an infected individual}\right) \times \beta \Delta t, \end{aligned}$$

where $\beta$ is the disease-specific transmission rate when a susceptible individual has contact with an infected individual. There are three kinds of infection to consider: (i) the infected person and the infector belong to the same community, (ii) an infected person visits a neighboring community and infects a resident of this community, and (iii) a susceptible person visits a neighboring community and gets infected by one of its residents. We will neglect the rarer “tourist to tourist” infections, when an infected person visits a neighboring community and infects there a visitor from yet another community. The term $\mathrm {Pr}\left( \text {meeting an infected individual}\right)$ can therefore be evaluated as a function of the pseudo-flux matrix $[f_{ab}]$ for each of the three aforementioned cases (SI). After summation of the three contributions, we obtain:

5 $$\begin{aligned} \beta _{aa} = \beta p, \qquad \beta _{ab} = \beta p \frac{f_{ab} + f_{ba}}{M_a}.\, \end{aligned}$$

where *p* is the frequency with which an individual is having contact with an other individual of its community, and is assumed to be the same for all communities. Equation (5) determines the infectivity matrix $[\beta _{ab}]$ from Eq. (1) up to a proportionality constant, namely $\beta p$.

## Results

### The model reproduces the spatial dynamics

**Figure 2:**
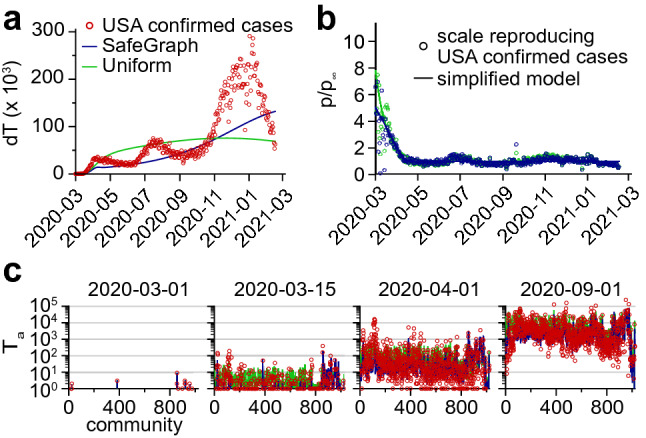
Model based on SafeGraph mobility data or a uniform infectivity matrix. (**a**) The model is fitted to COVID-19 confirmed cases in the USA. There is one fitting parameter per day. (**b**) Fitting parameters obtained. The shape suggests a simplified model with two limiting values before and after lockdown. (**c**) The simplified models obtained reproduce the spread of the SARS-CoV-2 epidemic in the community network. A direct comparison between the local epidemic sizes predicted by the models and the reported values can be found in Fig. 5. See also Movies S1 and S2.

In order to assess the validity of the infectivity matrix based on mobility data, Eq. (5), we confronted the model’s predictions against COVID-19 case numbers reported in the USA by the Center for Systems Science and Engineering (CSSE) at Johns Hopkins University^37^. Specifically, we fitted the daily $\beta p(t)$ values so as to minimize the sum of squared errors between values predicted by the model and values reported by the CSSE (Material and methods). The fitted $\beta p(t)$ values show a steep decay during the month of March 2020, followed by a plateau lasting until February 2021 (Fig. 2b).

### A two-phase simplified model

The time variations of $\beta p(t)$ shown in Fig. 2b imply a drastic decrease in the interaction frequency among individuals across all communities. This result reflects the effect of the shelter-in-place policies that were implemented at the beginning of the SARS-CoV-2 pandemic in many USA states. We can use this observation to estimate the effect of these policies quantitatively: the interaction frequency among individuals, namely *p*, is about 5 times smaller in the plateau following shelter-in-place policies than it was at the onset of the SARS-CoV-2 pandemic. Following this observation, we defined a simplified model, in which the fit with reported cases was carried out while enforcing a softplus shape for $\beta p(t)$ (Fig. 2b and Material and methods). This simplified model reproduced the average progression of the epidemic (Fig. 2a), but it didn’t capture the three oscillations visible in the number of new cases. We conclude that these oscillations in the number of new cases were mostly driven by a similar oscillatory pattern in the interaction frequency as seen in Fig. 2b. In Fig. 2c, we show for a few dates an overlay of the number of new COVID-19 cases in each community according to the official reports and as predicted by our simplified model. Despite non-negligeable variance, we find that the model reproduces to a satisfying degree the real dynamics. This can also be seen through a direct comparison of the model predictions with the real reports of new cases, for all dates combined, as shown in Fig. 5a. This result suggests that the infectivity matrix constructed from mobility data (Eq. 5) is a reasonable approximation of the “true” infectivity matrix.

### Turning down long-range interactions

**Figure 3:**
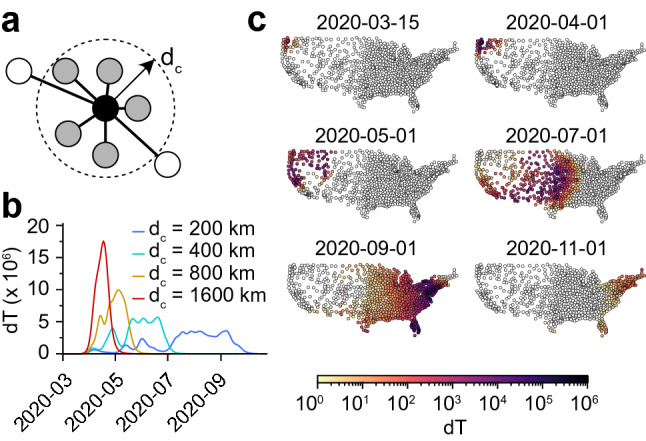
Limiting long-distance travels without local lockdown. (**a**) Only transmission rates for communities separated by a distance $d_{ij} < d_c$ are retained. (**b**) Daily new infections for increasing values of the cutoff distance. (**c**) Spatial visualization of the daily new infections using a cutoff $d_c = {200}\;{\text {km}}$. See also Movie S3.

The previous results suggest that the decrease in new COVID-19 cases was mostly driven by a country-wide reduction in the interaction frequency among individuals. Here we investigate the hypothetical effect of an alternative policy, namely a travel restriction while keeping unchanged the interaction frequency among individuals. Specifically, we modified the infectivity matrix so that communities separated by a physical distance larger than a prescribed cutoff do not interact: $\beta _{ij} = 0$ if $d_{ij} > d_c$ (Fig. 3a). We seeded the infection in a community belonging to the state of Washington and simulated the spread of the epidemics using a fixed interaction frequency ($\beta p(0)$ from the simplified model). As expected, we observed a reduction in the number of daily new cases *dT* (we define the local variables $dT_a(t) = T_a(t)-T_a(t-1)$) when the cutoff distance $d_c$ decreased, illustrating the “curve-flattening” effect that was targeted by travel restriction policies (Fig. 3b). In this idealized scenario with a single seed for the infection, the epidemic propagates as a traveling wave from the west coast to the east coast (Fig. 3c). However, as can be seen by comparing Fig. 2a to Fig. 3b, travel restriction policies are not as efficient as lockdown policies to decrease the spread of an epidemic.

### Properties of infectivity matrices

**Figure 4:**
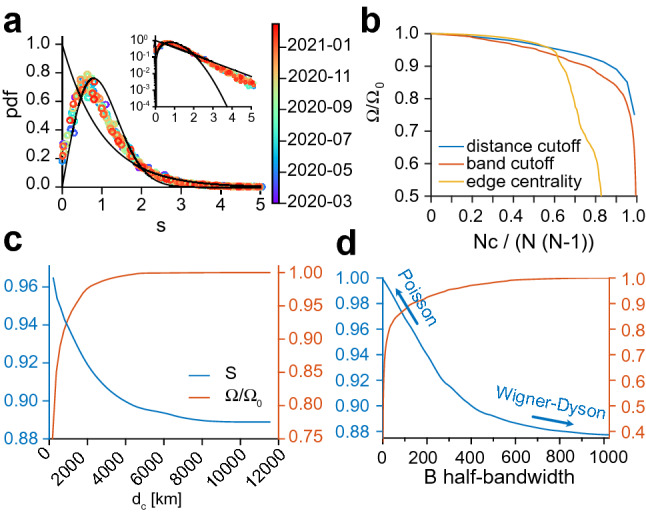
Random-matrix properties and mitigation strategies. (**a**) Level spacing distribution (unfolded^38,45–47^) of infectivity matrices between 2020-03-01 and 2021-02-15 (pooled by 7-day windows). The solid lines denote the Wigner-Dyson and the Poisson statistics. The level distribution is shown in Fig. S1. (**b**) The epidemic size $\Omega /\Omega _0$ versus the relative number of cuts $N_c/N/(N-1)$ for the 3 mitigation methods: distance cutoff, bandwidth, and edge-betweenness centrality. $\Omega _0$ is the epidemic size for $N_c=0$. Level-spacing entropy *S* and epidemic size as a function of (**c**) the cutoff distance $d_c$ in the range 170-12,000 km, and (**d**) the half-bandwidth $1{\le }B<1023$.

The daily infectivity matrices constructed from the SafeGraph mobility data can be viewed as elements of a random-matrix ensemble. Remarkably, matrix elements seem to be distributed according to the law $\beta _{ij} = \langle \beta _{ij} \rangle e^{\xi }$, where $\langle \beta _{ij} \rangle$ is the mean infectivity matrix and $\xi \equiv N(0,1)$ is a centered reduced Gaussian variable (Fig. S1). The probability density of the eigenvalues is also shown in Fig. S1. A connection can be made with random matrix theory (RMT)^38–40^, initially introduced by E. Wigner to model the spectra of the nuclei of heavy atoms, where the interactions between many nucleons are assumed to be drawn from a random ensemble. In RMT, random matrices are classified according to their symmetry, or according to their corresponding level statistics (namely, the probability density of the spacing between consecutive eigenvalues) that exhibit different degrees of level repulsion^38^. In particular, the Wigner-Dyson (WD) statistics is typical of the Gaussian Orthogonal Ensemble (in which eigenvalues “interact”), while the Poisson statistics is typical of a matrix with independent eigenvalues. The level-spacing statistics of the infectivity matrices is shown in Fig. 4a. We found that they were time-independent. Yet interestingly, the level statistics interpolates between the WD statistics and the Poisson statistics^41^. As shown earlier, removing links between communities according to their geographical distance results in a slower spread and a smaller epidemic size. Concomitantly, the level spacing distribution converges toward a Poisson statistics (Fig. 4c). The crossover from WD (entropy $S=0.7169$) to Poisson ($S=1$) distribution as links between communities are successively removed suggests an isolation policy that can lead to an effective reduction of the epidemic size (SI). As alternative mitigation strategies, we choose to induce the transition toward a Poisson distribution by decimating links between communities according to their “nominal” distance or the “edge-betweenness” centrality^42–44^ (Fig. 4b). Figure 4d shows how the level spacing distribution converges toward a Poisson statistics when the nominal distance threshold is lowered. We find the edge-betweenness centrality to be more efficient in decreasing the epidemic size. This is because decimation according to edge-betweenness first targets links with the largest transmission rates. One could also consider a moderate policy: instead of eliminating links completely, one could impose constraints on the flux of individuals that are allowed to commute via central pre-determined links.

### Classes of infectivity matrices

**Figure 5:**
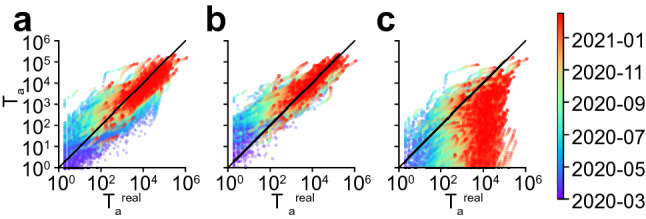
Comparison of the (fitted) model predictions with reported values using (**a**) the infectivity matrix derived from SafeGraph fluxes (same model as Fig. 2), (**b**) an infectivity matrix with uniform transmission rates, and (**c**) the infectivity matrix derived from SafeGraph fluxes truncated to geographical distances $d_c < {400} \; \text{km}$. Local epidemic sizes $T_a$ are shown. One symbol is associated to one given community and one given day.

Although SafeGraph mobility data^36^ provide a realistic picture of people movement between communities, one might ask what is the sensitivity of the results to the infectivity matrix derived from the mobility fluxes. We thus considered a model with uniform transmission rates among communities, namely $\beta _{ab} = \beta$, which suppresses spatial effects. In particular, this model leads to the natural variables $\nu _a$ (see SI) to be uniform: $\nu _a(t) = \nu ^\text {UN}(t)$. Surprisingly, carrying out the same fit to the reported cases (Fig. 1a,b) was only marginally inferior to the fit carried out with the SafeGraph mobility data (see Fig. 5b). By contrast, carrying out the same fit with the infectivity matrix derived from SafeGraph mobility data but with long-range interactions turned down resulted in a significantly different dynamics (see Fig. 5c). There are several implications of this result. (1) Although changes in the structure of the infectivity matrix can lead to drastically different dynamics (Figs. 3, 4 and 5c), it appears that the infectivity matrix derived from SafeGraph fluxes falls in the same “universality class” as the uniform model. This suggests that long-range movements (e.g. air traffic) played a prevalent role in the spread of SARS-CoV-2. (2) Our choice to reduce the complexity of the model to only one fitting parameter might not be adequate to discriminate between models falling into the same universality class. Instead of fitting only one scalar *p*(*t*) at each time point, we also investigated the possibility of fitting the $N^2$ transmission rates $\beta _{ab}(t)$ minimizing the errors with reported cases (see Material and methods and Fig. S2). Although this latter approach is clearly prone to overfitting, it shows that there exists a parametrization of the model which reproduces very closely reported cases. We anticipate that the proper number of fitting parameters lies in between those two extreme scenarios.

### Analysis of traveling waves

The results from the previous sections suggest the apparition of a wavefront when transmissions are short range. To investigate this phenomenon, we consider a simplified model of communities lying on a two-dimensional square lattice. Each community index is replaced by coordinates $(i,j) \in \llbracket 1, 2^n \rrbracket \times \llbracket 1, 2^m \rrbracket$. In particular, we consider that only individuals from neighboring communities interact together. We rewrite Eq. (1):

6 $$\begin{aligned} \frac{\mathrm {d}S_{i,j}}{\mathrm {d}t } = - S_{i,j} \left[ \alpha I_{i,j} + \beta ( I_{i+1,j} + I_{i-1,j} + I_{i,j+1} + I_{i,j-1} ) \right] , \quad \frac{\mathrm {d}I_{i,j}}{\mathrm {d}t } = -\frac{\mathrm {d}S_{i,j}}{\mathrm {d}t } - \gamma I_{i,j}, \quad \frac{\mathrm {d}R_{i,j}}{\mathrm {d}t} = \gamma I_{i,j}, \end{aligned}$$

where $\alpha$ (respectively $\beta$) is the intra-community (resp. inter-community) transmission rate. After rescaling the time and space variables, and defining the rescaled recovery rate $\tilde{\gamma } = \gamma / (a \beta )$ where $a = 4 + \alpha /\beta$, we look for wave solutions in the continuum limit by introducing the shape functions $S(x,y,t) = g(x - \tilde{v} t)$ and $I(x,y,t) = h(x - \tilde{v} t)$, where $\tilde{v} = v / \sqrt{a} \beta$ represents the velocity of the wave in rescaled time and space (see SI). The shape functions satisfy the ODE:

7 $$\begin{aligned} \begin{aligned} h''&= - \frac{\tilde{v}}{g} h' + \left( \frac{\tilde{\gamma }}{g} - 1 \right) h, \\ g'&= - f + \frac{\tilde{\gamma }}{\tilde{v}} h. \end{aligned} \end{aligned}$$

We find that the velocity of the traveling wave is bounded from below (SI):

8 $$\begin{aligned} \tilde{v} \ge \tilde{v}_c = 2 \sqrt{1 - \tilde{\gamma }}, \end{aligned}$$

which is in agreement with previous reports^18,48^ and with results from marginal-stability analysis^21,49,50^. Interestingly, by an independant argument, we also established (see SI) that $\tilde{v} \le \tilde{v}_c$. Therefore the traveling wave must move at velocity $\tilde{v} = \tilde{v}_c$. This indicates that the SIR dynamics in Eq. (6) falls into the Fisher-Kolmogorov-Petrovsky-Piscunov universality class, resulting in pulled waves^51–54^. Although we have taken the continuum limit of a nearest-neighbor model, this analysis is also valid for any finite-range infection matrix with the appropriate rescaling of variables.

**Figure 6:**
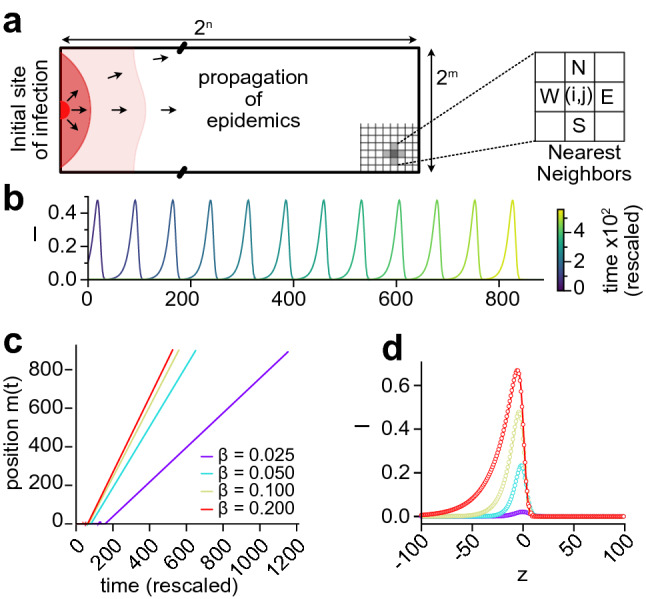
Existence of a wave with nearest-neighbors-only interactions. (**a**) Equation (6) is solved on a square lattice of $2^n \times 2^m$ sites. (**b**) Traveling front of infected individuals moving along the *x* direction (left to right). We took $\alpha = \beta = \gamma = {0.1}$, so that the rescaled recovery rate is $\tilde{\gamma } = \gamma / (a \beta ) = {0.2}$. (**c**) The dynamics simulated for different values of $\beta$. The position *m*(*t*) ($S(t, m(t))=(1 + S_\infty )/2$) of the wave is asymptotically linear in time, hence a constant velocity $\tilde{v} = \sqrt{1-\tilde{\gamma }}$. (**d**) The simulated wave profiles (symbols) are in agreement with the profiles predicted by Eq. (7) (solid lines). The corresponding S,I,R profiles are shown in Fig. S3. A simulation with $\tilde{\gamma } = {0.2}$ is shown in Movie S4.

We performed simulations of the dynamics given by Eq. (6) on a square lattice with a varying aspect ratio (Fig. 6a) and seeding the infection at one site on the west boundary. As expected, there is a front of new infections, moving from west to east as time progresses. A timelapse of a traveling front of infected individuals with $\alpha = \beta = \gamma = {0.1}$ is shown in Fig. 6b. The wave position increases asymptotically linearly with time, but the velocity $\tilde{v}$ of the wave varies with $\tilde{\gamma }$ (Fig. 6c). The profiles obtained are in agreement with the shape functions obtained by solving Eq. (7), as shown in Fig. 6d.

## Discussion

Biological systems are inherently complex and it can be a challenge to characterize them by a small number of parameters. In the case of epidemics, the huge number of parameters (for example, intercommunity spread involves $N^2$ transmission rates for *N* communities) can obscure the salient mechanisms and make it difficult for policy makers to find efficient interventions. Reducing the dimension of a model, when possible, is therefore of great value. In this article, we have proposed a spatial SIR model with an infectivity matrix based on the local travel patterns.

Despite its simplicity (there is only one global parameter to be adjusted), we find that our model is able to capture the spatial spread of the SARS-CoV-2 epidemic, with a delocalized multicenter spreading caused by long-distance travelers bringing infection into far regions, then becoming secondary centers of infection. The time evolution of the interaction frequency reflects the shelter-in-place policies that were implemented by various USA states in the early stages of the SARS-CoV-2 pandemic. In fact, the rich diversity of human responses could be summarized by a simplified model for the interaction frequency, whose asymptotes represent the values “before lockdown” and “after lockdown”, with few assumptions about the infection process.

If a model with relatively few parameters describes the observations, one can assume that it also describes the situation when these parameters are changed by an intervention. Therefore, we suggest such a model could be used as the basis for efficient policies—or at least reliably to estimate the consequences of adopting policies. As an example, we have shown the hypothetical effect of an alternative to the shelter-in-place policy in the case of the SARS-CoV-2 pandemic. Specifically, we have investigated a travel restriction policy in which individuals can only move within an area of fixed radius centered around their residence. By contrast to the lockdown policy, we find that infections spread through a well-defined wave front, traveling with a certain velocity. This scenario might be preferable since it gives time to communities and public health infrastructures to prepare for the onset of the epidemics, while still “flattening the curve” of new infections (Fig. 3b). We have also provided both an analytical and numerical analysis elucidating the mechanism of formation of a wavefront. In particular, we give the velocity and the shape of the wavefront for an epidemic spreading through nearest neighbors interactions.

During the course of our research, an epidemiological study bearing similarities with our approach was published^55^. In that study, the authors developed a county-resolved metapopulation model describing the spread of SARS-CoV-2 in the USA, informed with mobility data from SafeGraph. Instead of calibrating the model by fitting the country-wide number of reported cases as in the present study, the authors fitted their model to reproduce reported cases of COVID-19 on a per-county basis. Furthermore, the model required fitting many time-dependent parameters on a per-county basis, including per-county transmission rates, making the fitting procedure very high dimensional. Finally, the dynamics of the disease spread introduced differs from Eq. (1) since *S*, *I* and *R* compartments were introduced for each commute channel $i \rightarrow j$ rather than for each community. Altogether, the complexity of that model makes it less amenable to analytical study.

In conclusion, we used a simple model of intercommunity spread of an infectious disease to show the transition between different regimes of epidemic progression. Because of the complexity of the infection process (e.g., variations in individuals’ responses, mutations, etc.) and of human behavior, we are still far from a global forecasting system able to predict the spread of different infectious agents throughout the world. As with weather forecasting, observables must be measured in real time in order to inform complex models to yield short-term forecasts. One salient feature in our approach is showing how such widespread measurements (namely, the mobility data) can be integrated in a model describing the spread of an infectious agent, and showing what types of predictions can be obtained. It is a step toward more predictive epidemiology models, grounded in measurable quantities.

## Material and methods

### Mobility data

The mobility data was obtained from SafeGraph, a company that aggregates anonymized location data from numerous applications in order to provide insights about physical places, via the SafeGraph Community. To enhance privacy, SafeGraph excludes census block group (CBG) information if fewer than two devices visited an establishment in a month from a given census block group. In this manuscript, we use data extracted from the “Social Distancing Metrics” dataset^36^, with dates between February 2020 and February 2021. SafeGraph has stopped sharing mobility data under this format, however the same data can be acessed under a different format through the Neighborhood Patterns dataset^56^. However the matrices of fluxes between our coarse-grained communities can be found in the ‘data’ folder in our github repository. We obtained the coarse-grained communities by running a *K*-means clustering algorithm to group the 220,333 CBGs into ${2^{10}}$ communities. We used the implementation from Scikit Learn. We then ran a hierarchical clustering algorithm to re-index communities so that communities close in space had close indices, as shown in Fig. 1. We used the linkage function from SciPy. We then computed for every day the “flux matrix” where each entry $f_{ab}$ represents the number of cell phone whose residence CBG belongs to community *a* which visited a CBG belonging to community *b*. The average flux matrix was constructed by averaging all the daily flux matrices. We used population counts for CBGs in agreement with the United States Census Bureau’s and available in the SafeGraph Open Census Data^57^ (file “cbg_b01.csv”, column “B01001e1”). We checked that the population counts from the United States Census Bureau were approximately proportional to the residential mobile-phone counts, therefore validating mobile tracking as a proxy for actual population.

### Reports on SARS-CoV-2 infections

In order to fit our model, we used USA cases of COVID-19 reported by the Center for Systems Science and Engineering (CSSE) at Johns Hopkins University^37^ which can be accessed at the GitHub repository github.com/CSSEGISandData/COVID-19. New cases of COVID-19 reported by the CSSE were mapped to the closest mobility-data-derived community based on their latitude and longitude. We therefore obtained the time evolution of SARS-CoV-2 infections through the mobility-data derived communities. Integer absolute new cases were converted into relative population fractions using the community population counts obtained from the United States Census Bureau.

### Model fit

#### Daily scales

Here we consider the SIR dynamics (see Supplementary Information):

9 $$\begin{aligned} \begin{aligned} \frac{\mathrm {d}s_a}{\mathrm {d}t}(t)&= - p(t) s_a(t) \sum \limits _b \beta _{ab} j_b(t), \\ \frac{\mathrm {d}j_a}{\mathrm {d}t}(t)&= p(t) s_a(t) \sum \limits _b \beta _{ab} j_b(t) - \gamma j_a(t). \end{aligned} \end{aligned}$$

We also define the chi-square at each time *t*:

10 $$\begin{aligned} \begin{aligned} \chi ^2(t)&= \sum \limits _{a=1}^N M_a^2 \left( s_a(t+1) - \hat{s}_a(t+1) \right) ^2, \end{aligned} \end{aligned}$$

where $M_a$ is the population at location *a* and $(1 - \hat{s}_a) M_a$ is the number of reported cases at location *a*. Assuming that $p(t-1), p(t-2), \ldots , p(0)$ have been previously evaluated, the dynamics $(s_a, j_a)$ is determined up to time *t*, and we have:

11 $$\begin{aligned} \begin{aligned} s_a(t+1)&= s_a(t) - p(t) \int \limits _t^{t+1} \mathrm {d}u \, s_a(u) \sum \limits _b \beta _{ab} j_b(u), \\ j_a(t+1)&= j_a(t) + p(t) \int \limits _t^{t+1} \mathrm {d}u \, \left( s_a(u) \sum \limits _b \beta _{ab} j_b(u) - \gamma j_a(u) \right) . \end{aligned} \end{aligned}$$

To obtain the scale *p*(*t*), we solve for:

12 $$\begin{aligned} p^\text {fit}(t) = \text {argmin}(\chi (t)^2). \end{aligned}$$

#### Simplified model

We look for a simplified model in which the scales have a functional form close to a ramp function:

13 $$\begin{aligned} \begin{aligned} p_\theta (t) = \theta _3 \ln {\left( 1 + e^{-\theta _1 (t-\theta _2)} \right) } + \theta _4. \end{aligned} \end{aligned}$$

We first set $\theta$ by fitting this function to the daily scales:

14 $$\begin{aligned} \theta ^*= \text {argmin}\left( \sum \limits _t (p_\theta (t) - p^\text {fit}(t))^2 \right) \end{aligned}$$

Then we adjust the slope of the ramp in order to minimize the error with reported cases. In particular, we set $\theta = (\theta _1^*, \theta _2^*, \psi , \theta _4^*)$, and we solve:

15 $$\begin{aligned} \psi ^*= \text {argmin} \left( \sum \limits _t \chi (t)^2\right) . \end{aligned}$$

We thus define $\theta ^\text {simplified} = (\theta _1^*, \theta _2^*, \psi ^*, \theta _4^*)$, and the model for simplified scales is:

16 $$\begin{aligned} p^\text {simplified}(t) = p_{\theta ^\text {simplified}}(t). \end{aligned}$$

#### Daily infectivity matrices

We also considered another approach, consisting of fitting the $N^2$ transmission rates $\beta _{ab}(t)$ at every time *t*. In particular, we solve:

17 $$\begin{aligned} \left\{ \beta _{ab}^\text {opt}(t) \right\} = \text {argmin}(\chi (t)^2), \end{aligned}$$

subject to the constraints $\beta _{ab} \ge 0$. We show the results of this fit in Fig. S2, however this approach is prone to overfitting since there are $N^2$ fitting parameters and only *N* data point at each time *t*.

### Simulations with nearest-neighbors-only interactions

The curves shown in Fig. 6b,c and the symbols shown in Fig. 6d were obtained by integrating Eq. (6).We used the function solve_ivp from SciPy with the “DOP853” integration method. At $t = 0$, we considered $S = 1$ everywhere except at the sites of coordinates $(0, 2^{m-1}-1)$ and $(0, 2^{m-1})$ (see Fig. 6a), where we set $S=0$ and $I = 1$. The Laplacian was computed using the 9-point stencil $$\Delta _\text {discrete} = \left( {\begin{matrix} 1 &{} 2 &{} 1 \\ 2 &{} -12 &{} 2 \\ 1 &{} 2 &{} 1 \end{matrix}}\right) /4$$. We considered periodic boundary conditions along the *y* direction and Dirichlet boundary conditions along the *x* direction.

## Supplementary Information

Supplementary Video S1.

Supplementary Video S2.

Supplementary Video S3.

Supplementary Video S4.

Supplementary Video S5.

Supplementary Video S6.

Supplementary Video S7.

Supplementary Information 8.

Supplementary Information 9.

## Data Availability

Data and scripts used in this study are available at the GitHub repository github.com/czbiohub/epidemiology_flux_model. Except the raw mobility data, which belongs to SafeGraph.
