# Supplementary material for: A high-resolution flux-matrix model describes the spread of diseases in a spatial network and the effect of mitigation strategies
Source: Sci Rep. 2022 Sep 24;12:15946. doi: 10.1038/s41598-022-19931-w (PMC9509367; doi:10.1038/s41598-022-19931-w)
Supplement: Supplementary file 8 — Supplementary Information 8. [file 41598_2022_19931_MOESM8_ESM.pdf]

Supplementary Information for :“A high-resolution flux-matrix model describes the spread of diseases in a spatial network and the effect of mitigation strategies”

Guillaume Le Treut*, **Greg Huber***, **Mason Kamb** , **Kyle Kawagoe** , **Aaron McGeever** , **Jonathan Miller** , **Reuven Pnini** , **Boris Veytsman** , **and David Yllanes**

*To whom correspondence should be addressed: guillaume.letreut@czbiohub.org, greg.huber@czbiohub.org.

Contents

1 Supplementary figures 2
2 Supplementary movies 5
3 Formal solution to the spatial SIR 6
4 Initial stage of an epidemic 8
5 Construction of the infectivity matrix from mobility data 8
6 Spreading with a wave of infection 9
7 Level statistics and connectedness 11

1 Supplementary figures

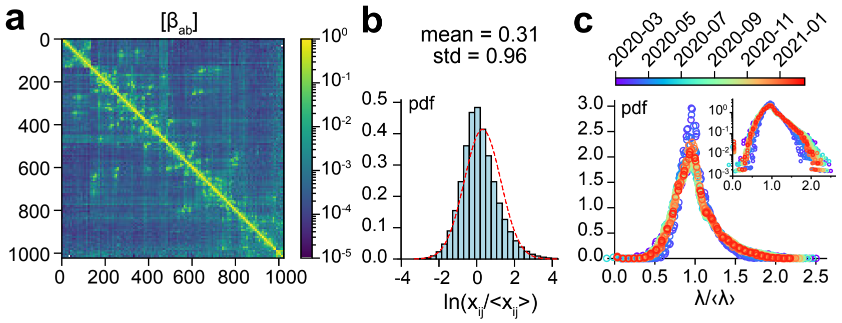

Figure S1. Supplementary figure to Figure 4 of the main text. **(a)** Mean infectivity matrix (between 2020-03-01 and 2021-02-15). Entries were pooled in squares of size 8×8. The maximum is shown. **(b)** The noise distribution suggests a log-normal distribution of each entry around its mean. **(c)** Eigenvalue distribution of infectivity matrices as a function of time (pooled by 7-day windows).

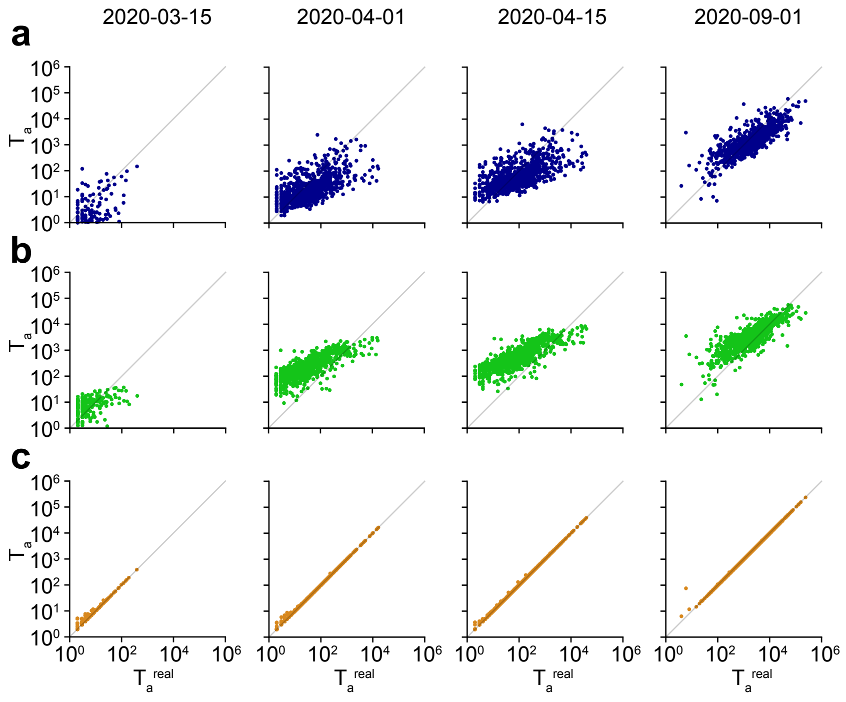

Figure S2. Comparison of instantaneous model predictions with reported values at different dates. **(a)** Scale fitting procedure with infectivity matrix derived from SafeGraph mobility data. **(b)** Scale fitting procedure with uniform infectivity. **(c)** Fitting procedure with N^2 transmission rates $\beta_{ab}(t)$.

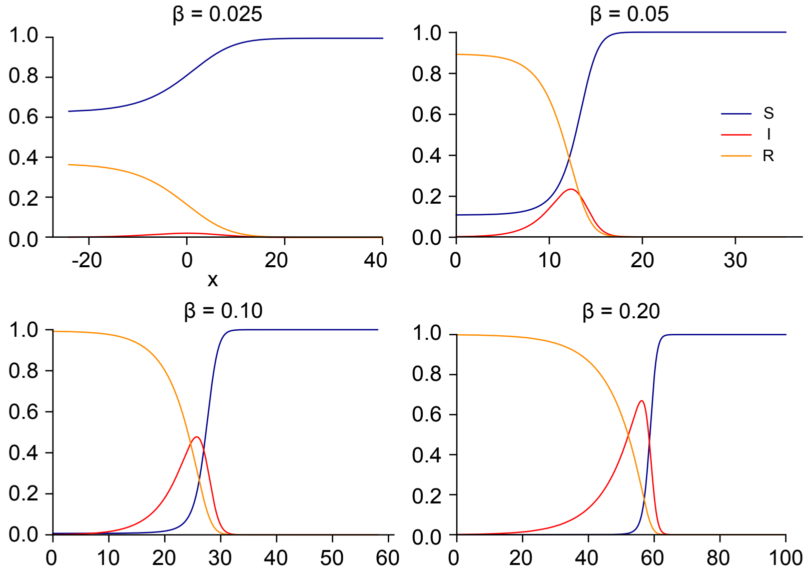

Figure S3. Supplementary figure to Figure 6 of the main text. Wave profiles for the values of β shown in Figure 6**d-e**.

2 Supplementary movies

Movie S1. Movie corresponding to Figure 2**c**.

Movie S2. Map of new COVID-19 cases per day per community (model from Figure 2).

Movie S3. Map of new COVID-19 cases per day per community when interactions are limited to nearest neighbors within a ball of radius $d_c = 200\,\mathrm{km}$ (model from Figure 3).

Movie S4. Traveling wave of infections for the spread of an infectious disease simulated on a 2d lattice with nearest-neighbors-only interactions (see Figure 6).

Movie S5. The flux matrix and the level spacing distribution as a function of the cutoff distance d_c (see Figure 4**c**).

Movie S6. The flux matrix and the level spacing distribution as a function of half-bandwidth B (see Figure 4**d**).

Movie S7. Edge-betweenness centrality and the level spacing distribution as a function of the relative number of cuts (see Figure 4**b**).

3 Formal solution to the spatial SIR

3.1 Matrix SIR

We consider N communities affected by a growing epidemic. The spreading of the epidemic is described by the multi-compartment SIR model. Every community a is characterized by the number of susceptible individuals S_a, the infected individuals I_a and the number of recovered individuals R_a. The dynamics for S_a, I_a and R_a with $a \in [\![1,N]\!]$ is described by the equations

$$\begin{aligned}\frac{\mathrm{d}S_a}{\mathrm{d}t} &= -S_a \sum_b \beta_{ab} \frac{I_b}{M_b},\\ \frac{\mathrm{d}I_a}{\mathrm{d}t} &= -\frac{\mathrm{d}S_a}{\mathrm{d}t} - \gamma I_a,\\ \frac{\mathrm{d}R_a}{\mathrm{d}t} &= \gamma I_a.\end{aligned} \tag{S1}$$

We assume that the population in each community is fixed, i.e.

$$S_a(t) + I_a(t) + R_a(t) = M_a = \text{const.} \tag{S2}$$

The boundary conditions for equation (S1) are:

$$\begin{aligned}S_a(0) &= (1-\varepsilon_a)M_a,\\ I_a(0) &= \varepsilon_a M_a,\\ R_a(0) &= 0,\end{aligned} \tag{S3}$$

where the parameter ε_a describes the initial infection (the initial number of infections per per person in the given community).

We introduce the rescaled variables: $s_a = S_a/M_a$, $j_a = I_a/M_a$ and $r_a = R_a/M_a$. Then equations (S1) and (S3) become

$$\begin{aligned}\frac{\mathrm{d}s_a}{\mathrm{d}t} &= -s_a \sum_b \beta_{ab} j_b,\\ \frac{\mathrm{d}j_a}{\mathrm{d}t} &= -\frac{\mathrm{d}s_a}{\mathrm{d}t} - \gamma j_a,\\ \frac{\mathrm{d}r_a}{\mathrm{d}t} &= \gamma j_a,\end{aligned} \tag{S4}$$

with

$$\begin{aligned}s_a(0) &= (1-\varepsilon_a),\\ j_a(0) &= \varepsilon_a,\\ r_a(0) &= 0.\end{aligned} \tag{S5}$$

3.2 Solution of matrix SIR equations: the canonical basis

In order to solve equations (S4) and (S5), we define:

$$\xi_a(t) = \sum_b \beta_{ab} j_b(t), \quad \nu_a(t) = \int_0^t \mathrm{d}u\, \xi_a(u). \tag{S6}$$

Substituting equation (S6) in equation (S4), we solve for s_a and obtain:

$$s_a(t) = (1-\varepsilon_a)e^{-\nu_a(t)}. \tag{S7}$$

We also note that $r_a(t)$ can be written as:

$$r_a(t) = \gamma \sum_b \beta^{-1}_{ab} \nu_a(t), \tag{S8}$$

where $[\beta_{ab}^{-1}]$ denotes the inverse matrix such that $\sum_c \beta_{ac}\beta_{cb}^{-1} = \delta_{ab}$. We therefore obtain the parametrization:

$$\begin{aligned}
s_a(t) &= (1-\varepsilon_a)e^{-\nu_a(t)},\\
j_a(t) &= 1-(1-\varepsilon_a)e^{-\nu_a(t)} - \gamma\sum_b \beta_{ab}^{-1}\nu_a(t),\\
r_a(t) &= \gamma\sum_b \beta_{ab}^{-1}\nu_a(t),
\end{aligned} \tag{S9}$$

as a function of $\nu_a(t)$. Note that when the infectivity matrix is diagonal, namely $\beta_{ab} = \beta\delta_{ab}$, we recover the parametrization from reference[4]:

$$\begin{aligned}
s(t) &= s(0)u,\\
j(t) &= \frac{\gamma}{\beta}\ln u - s(0)u + 1,\\
r(t) &= -\frac{\gamma}{\beta}\ln u,
\end{aligned} \tag{S10}$$

with $u(t) = e^{-\nu(t)}$.

The dynamics is determined by the functions $\nu_a(t)$. Starting from equation (S4), we multiply the equations by β_{ab} and sum, obtaining

$$\begin{aligned}
\frac{\mathrm{d}}{\mathrm{d}t}\left(\sum_b \beta_{ab} j_b\right) + \gamma\sum_b \beta_{ab} j_b = \sum_b \beta_{ab}(1-\varepsilon_b)\frac{\mathrm{d}\nu_b}{\mathrm{d}t}e^{-\nu_b} &\Leftrightarrow\\
\frac{\mathrm{d}\xi_a}{\mathrm{d}t} + \gamma\xi_a = \sum_b \beta_{ab}(1-\varepsilon_b)\frac{\mathrm{d}}{\mathrm{d}t}\left(-e^{-\nu_b}\right) &\Leftrightarrow\\
\xi_a(t) - \xi_a(0) + \gamma\nu_a(t) = -\sum_b (1-\varepsilon_b)\left(e^{-\nu_b}-1\right) &\Leftrightarrow\\
\frac{\mathrm{d}\nu_a}{\mathrm{d}t} + \gamma\nu_a = \sum_b \beta_{ab}\left(1-(1-\varepsilon_b)e^{-\nu_b}\right)&.
\end{aligned} \tag{S11}$$

Equation (S11) can be solved numerically. In the $t\to\infty$ limit, we obtain:

$$\gamma\nu_a(\infty) = \sum_b \beta_{ab}\left(1-(1-\varepsilon_b)e^{-\nu_b(\infty)}\right), \tag{S12}$$

which is the same as setting $j_a = 0$ in equation (S9). Equation (S12) can be solved iteratively. The size of the epidemic is given by:

$$\begin{aligned}
\Omega(t) &= \sum_a \left(I_a(t) + R_a(t)\right),\\
&= \sum_a M_a\left(1-(1-\varepsilon_a)e^{-\nu_a(t)}\right),\\
&\xrightarrow[t\to\infty]{} \sum_a M_a\left(1-(1-\varepsilon_a)e^{-\nu_a(\infty)}\right).
\end{aligned} \tag{S13}$$

3.3 Solution of matrix SIR equations: SVD basis

A solution can also be written using the singular value decomposition of the infectivity matrix β:

$$\beta_{ab} = \sum_\alpha \lambda_\alpha v_{\alpha a} u_{\alpha b}. \tag{S14}$$

For a given vector $\mathbf{x}$, we use the following notation: $x_\alpha = \sum_a v_{\alpha a} x_a$. From equation (S11) we thus obtain:

$$\frac{\mathrm{d}\nu_\alpha}{\mathrm{d}t} + \gamma\nu_\alpha = \lambda_\alpha \sum_a u_{\alpha a}\left(1-(1-\varepsilon_a)e^{-\sum_\tau v_{\tau a}\nu_a}\right). \tag{S15}$$

4 Initial stage of an epidemic

We again start from equation (S4), which we linearize around the initial state $s_a^{(0)} = 1$, $j_a^{(0)} = 0$, and $r_a^{(0)} = 0$. To first order $\mathbf{j}$ and $\mathbf{r}$ satisfy the ODE:

$$\begin{aligned} \frac{\mathrm{d}j_a}{\mathrm{d}t} &= \sum_b \beta_{ab} j_b - \gamma j_a, \\ \frac{\mathrm{d}r_a}{\mathrm{d}t} &= \gamma j_a. \end{aligned} \tag{S16}$$

$[\beta_{ab}]$ is a real square positive matrix. According to the Perron-Frobenius theorem, there exists the maximal eigenvalue $\lambda_\omega > 0$, such that any other eigenvalue $\lambda_\alpha < \lambda_\omega$, and the associated eigenvector $\mathbf{v}_\omega$ is positive. At large times, we have:

$$\mathbf{j} \sim (\mathbf{j}(0) \cdot \mathbf{v}_\omega)\, e^{(\lambda_\omega - \gamma)t} \mathbf{v}_\omega, \tag{S17}$$

where $(\mathbf{A} \cdot \mathbf{B})$ denotes a scalar product. The epidemic grows only if the basic reproduction number $\mathscr{R}_0 = \frac{\lambda_\omega}{\gamma} > 1$. Furthermore, if the infectivity matrix can be factorized as $\beta_{ab} = f_a g_b$, then β is of rank 1 and λ_ω is the only non-zero eigenvalue. We then have $\lambda_\omega = \sum_a f_a g_a$ and $\mathbf{v}_\omega = \mathbf{f}$.

5 Construction of the infectivity matrix from mobility data

The mobility data obtained from SafeGraph allows us to construct a pseudo-flux matrix in which each entry f_{ab} represents the number of individuals from community a visiting community b per day. Let us now consider one community a, having S_a susceptible individuals and I_a infected individuals. The variation in susceptible individuals due to new infections during the time interval Δt has the form:

$$S_a(t + \Delta t) - S_a(t) = -S_a \times \Pr(\text{meeting an infected individual}) \times \beta \Delta t, \tag{S18}$$

where $\beta \Delta t$ represents the probability to get infected when meeting an infected individual. We now list the different contributions.

5.1 Intra-community contributions

The contributions coming from infected individuals in the same community are:

$$\begin{aligned} & S_a \times p_a \frac{I_a}{\sum_b f_{ba} + M_a} \times \beta \Delta t, \\ & = p_a \beta \Delta t S_a I_a \frac{1}{M_a} \left(1 - \frac{f_{*a}}{M_a}\right) + \mathrm{o}\left(\frac{f_{*a}}{M_a}\right), \end{aligned} \tag{S19}$$

where we have introduced $f_{*a} = \sum_b f_{ab}$, and the parameter p_a representing the frequency with which an individual is interacting with other individuals in community a. Here $I_a / (f_{*a} + M_a)$ is the probability to meet an infected individual when interacting with an individual in the community.

5.2 Inter-community contributions: incoming visitors

The contributions to the infections in community a from infected individuals visiting from another community b are:

$$\begin{aligned} & S_a \times p_a \frac{f_{ba}}{f_{*a} + M_a} \frac{I_b}{M_b} \times \beta \Delta t, \\ & = p_a \beta \Delta t S_a I_b \frac{f_{ba}}{M_a M_b} \left(1 - \frac{f_{*a}}{M_a}\right) + \mathrm{o}\left(\frac{f_{*a}}{M_a}\right), \end{aligned} \tag{S20}$$

where we have assumed that $f_{ba} I_b / M_b$ is the number of infected visitors from b.

5.3 Inter-community contributions: returning natives

The contributions coming from individuals from community a infected while visiting another community b are:

$$\begin{aligned} & f_{ab} \frac{S_a}{M_a} \times p_b \frac{I_b}{f_{*b} + M_b} \times \beta \Delta t, \\ & = p_b \beta \Delta t S_a I_b \frac{f_{ab}}{M_a M_b} \left(1 - \frac{f_{*b}}{M_a}\right) + \mathrm{o}\left(\frac{f_{*b}}{M_a}\right), \end{aligned} \tag{S21}$$

where we have assumed that $f_{ab}S_a/M_a$ is the number of susceptible visitors from a visiting community b.

Neglecting the f_{*a}/M_a terms, we obtain after adding all contributions:

$$\frac{\mathrm{d}S_a}{\mathrm{d}t} = -S_a \sum_b \beta_{ab} \frac{I_b}{M_b}, \tag{S22}$$

with:

$$\begin{aligned} \forall a \in [\![1,N]\!], \quad \beta_{aa} &= \beta p_a, \\ \forall a < b, \quad \beta_{ab} &= \beta \frac{f_{ab}p_b + f_{ba}p_a}{M_a}. \end{aligned} \tag{S23}$$

Note that in general, the matrix $[\beta_{ab}]$ is not symmetric. In the manuscript, we make the assumption that the interaction frequency is the same in all communities, namely $p_a = p$.

6 Spreading with a wave of infection

6.1 ODE for the wave profile

We consider the SIR model on a 2d lattice, with infections limited to nearest-neighbors, and with uniform population $M_a = M$. For the sake of simplicity, we will consider the rescaled S, I, R variables with $S+I+R = M = 1$. We write:

$$\begin{aligned} \frac{\mathrm{d}S_{i,j}}{\mathrm{d}t} &= -S_{i,j}\left(\alpha I_{i,j} + \beta\left(I_{i-1,j} + I_{i+1,j} + I_{i,j-1} + I_{i,j+1}\right)\right), \\ \frac{\mathrm{d}I_{i,j}}{\mathrm{d}t} &= -\frac{\mathrm{d}S_{i,j}}{\mathrm{d}t} - \gamma I_{i,j}, \\ \frac{\mathrm{d}R_{i,j}}{\mathrm{d}t} &= \gamma I_{i,j}, \end{aligned} \tag{S24}$$

where i and j denote the indices along the first and second dimensions. We introduce the discrete laplacian:

$$\Delta I_{i,j} = \frac{\left(I_{i-1,j} + I_{i+1,j} - 2I_{i,j}\right) + \left(I_{i,j-1} + I_{i,j+1} - 2I_{i,j}\right)}{l^2},$$

where l is the lattice spacing. Equation (S24) becomes:

$$\begin{aligned} \frac{\mathrm{d}S_{i,j}}{\mathrm{d}t} &= -\beta S_{i,j}\left(aI_{i,j} + l^2 \Delta I_{i,j}\right), \\ \frac{\mathrm{d}I_{i,j}}{\mathrm{d}t} &= -\frac{\mathrm{d}S_{i,j}}{\mathrm{d}t} - \gamma I_{i,j}, \\ \frac{\mathrm{d}R_{i,j}}{\mathrm{d}t} &= \gamma I_{i,j}, \end{aligned} \tag{S25}$$

where $a = 4 + \alpha/\beta$. In this study, we will consider $\alpha = \beta$, so $a = 5$. In the continuum we have:

$$\begin{aligned} \partial_t S &= -\beta S\left(aI + l^2(\partial_x^2 + \partial_y^2)I\right), \\ \partial_t I &= -\partial_t S - \gamma I, \\ \partial_t R &= \gamma I, \end{aligned} \tag{S26}$$

Limiting ourselves to solutions which only depend on one space variable, namely $S(t,x,y) = S(t,x)$, and after rescaling the time variable, $t \leftarrow t(a\beta)^{-1}$, the space variable, $x \leftarrow xla^{-1/2}$, and the recovery rate, $\gamma \leftarrow (a\beta)\gamma$, we obtain:

$$\begin{aligned} \partial_t S &= -S\left(I + \partial_x^2 I\right), \\ \partial_t I &= S\left(I + \partial_x^2 I\right) - \gamma I. \end{aligned} \tag{S27}$$

We are interested in the traveling front solutions, namely $S(x,t) = g(x - vt)$ and $I(x,t) = h(x - vt)$, where v is the velocity of the traveling waves. Here, $g(z)$ and $h(z)$ are the shape functions for the propagating front. Making those substitutions in equation (S27), we obtain the ODE:

$$\begin{aligned} f' &= -\frac{v}{g} f + \left(\frac{\gamma}{g} - 1\right) h, \\ h' &= f, \\ g' &= -f + \frac{\gamma}{v} h. \end{aligned} \tag{S28}$$

6.2 Velocity selection

The solution to equation (S28) specifies the shape functions g and h for a traveling front solution with velocity v. We now characterize what are the admissible velocities. We start by computing the jacobian of the function on the right-handside of equation (S28):

$$J = \begin{pmatrix} -\frac{v}{g} & \frac{\gamma}{g}-1 & -\frac{\gamma h - vf}{g^2} \\ 1 & 0 & 0 \\ -1 & \frac{\gamma}{v} & 0 \end{pmatrix}, \tag{S29}$$

and its characteristic polynomial:

$$\begin{aligned} P(\lambda) &= \det(J-\lambda I), \\ &= -\lambda^2\left(\lambda+\frac{v}{g}\right)+\left(\lambda-\frac{\gamma}{v}\right)\frac{\gamma h - vf}{g^2}+\lambda\left(\frac{\gamma}{g}-1\right). \end{aligned} \tag{S30}$$

The fixed points of the ODE in equation (S28) are of the form $(0,0,g)$, with $g\in[0,1]$. At those points, there is one zero eigenvalue, and two eigenvalues satisfying the equation:

$$\lambda + \frac{1-\gamma/g}{\lambda} = -\frac{v}{g}. \tag{S31}$$

The fraction of susceptible individuals $S(x,t)$ is a decreasing function of t. Thus it forbids any oscillatory behavior in the shape function $g(z)$. Therefore the two eigenvalues must be real, which results in the condition:

$$\Delta = \frac{v^2}{g^2} - 4\left(1-\frac{\gamma}{g}\right) \geq 0 \quad \Leftrightarrow \quad v \geq 2g\sqrt{1-\frac{\gamma}{g}}. \tag{S32}$$

The function in the right-handside of the inequality is an increasing function of g. Furthermore, the fixed point $(0,0,1)$ must belong to the front solution since it corresponds to the unstable initial condition with only susceptible individuals. Therefore, enforcing that all admissible fixed points have real eigenvalues yields the condition:

$$v \geq v_c = 2\sqrt{1-\gamma}. \tag{S33}$$

6.3 Upper bound on the velocity

Since $S\leq 1$, by retracing the steps from equation (S24) to equation (S27) we have that:

$$\partial_t I \leq \partial_x^2 I + (1-\gamma)I. \tag{S34}$$

Let us define $\tilde{I}$, which is a solution to

$$\partial_t \tilde{I} = \partial_x^2 \tilde{I} + (1-\gamma)\tilde{I}. \tag{S35}$$

Starting from the same initial condition, $I(0,x)=\tilde{I}(0,x)$, we must have at all times:

$$I(x,t) \leq \tilde{I}(x,t). \tag{S36}$$

The function $\phi(x)=(1-\gamma)x$ satisfies the condition[51] $\phi'(x)\leq\phi'(0)$ for all $x\in[0,1]$, therefore equation (S35) falls into the Fisher-Kolmogorov-Petrovsky-Piscunov (FKPP) universality class[51–54]. In particular, for a step initial condition, $\tilde{I}(t,x)$ evolves into a moving front with velocity $v=v_c=2\sqrt{\phi'(0)}=2\sqrt{1-\gamma}$.

Because of the inequality in equation (S36), any moving front I must move at a velocity smaller than the velocity of the moving front $\tilde{I}$. Otherwise, the I front would eventually pass the $\tilde{I}$ front, which violates equation (S36). Therefore, the velocity of the I front must satisfy:

$$v \leq v_c. \tag{S37}$$

6.4 Pulled wave

Combining equations (S33) and (S37), we obtain that any moving front resulting from equation (S27) moves at velocity $v=v_c=2\sqrt{1-\gamma}$. Therefore it falls in the FKPP universality class and it is a pulled wave.

We have solved numerically equation (S27) for several values of γ, starting from the initial condition: $S(0,x)=1, I(0,x)=0, \forall x>0$ and $S(0,0)=0, I(0,0)=1$. In Figure S4**a**, we show the position of the moving front as a function of time. The position of the moving front, $m(t)$, was defined such that $S(t,m(t))=(1+S_\infty)/2$. Clearly, after a transient regime, the solution evolves into a moving front. We plotted the velocity v as a function of γ in Figure S4**b**, which agrees with the theoretical prediction $v=v_c=2\sqrt{1-\gamma}$.

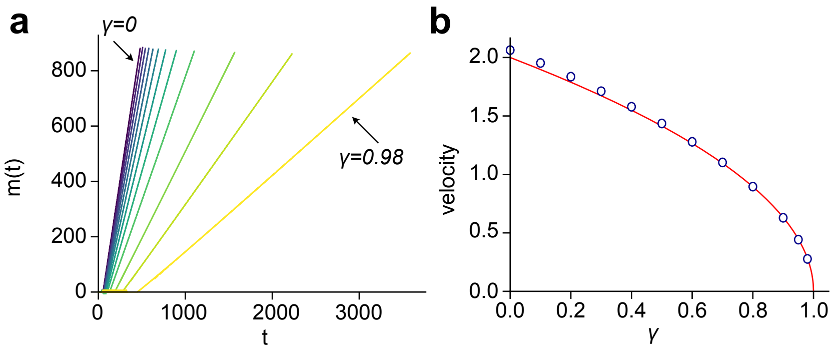

Figure S4. Traveling wave solutions to equation (S27). **(a)**. Position of the moving front as a function of time, $m(t)$, for values of $\gamma = 0, 0.1, 0.2, 0.3, 0.4, 0.5, 0.6, 0.7, 0.8, 0.9, 0.95, 0.98$. **(b)** Fitted velocities collapse on the theoretical $v = v_c$.

6.5 Shape of the traveling front

Since a traveling front solution must travel at velocity v_c, it follows that the shape is uniquely determined by the recovery rate γ. The shape functions g and h satisfy the ODE:

$$\begin{aligned} f' &= -\frac{2\sqrt{1-\gamma}}{g} f + \left(\frac{\gamma}{g} - 1\right) h, \\ h' &= f, \\ g' &= -f + \frac{\gamma}{2\sqrt{1-\gamma}} h. \end{aligned} \tag{S38}$$

The shape of the traveling front must be such that:

$$\begin{cases} \lim_{z\to-\infty} g(z) = \lim_{t\to+\infty} S(t,x) = S_\infty, \\ \lim_{z\to-\infty} h(z) = \lim_{t\to+\infty} I(t,x) = 0. \end{cases} \quad \text{and} \quad \begin{cases} \lim_{z\to+\infty} g(z) = S(0,x) = 1, \\ \lim_{z\to+\infty} h(z) = I(0,x) = 0, \end{cases} \tag{S39}$$

We determine the residual fraction of susceptible individuals, S_∞, using the parametrization in equation (S10) with $u = e^{-\nu}$, and solving:

$$1 = e^{-\nu_\infty} + \gamma \nu_\infty. \tag{S40}$$

Since the last equation is solved numerically, it is useful to derive two bounds. First, the condition $S_\infty > 0$ yields the condition $\nu_\infty < \nu^{**} = 1/\gamma$. Second, the function $e^{-x} + \gamma x - 1$ is strictly negative in the interval $]0, \nu_\infty[$, with a minimum reached at $\nu^* = -\ln \gamma$. Thus we have $\nu^* < \nu_\infty < \nu^{**}$.

Following the stability analysis introduced hereabove, $(0,0,g)$ is an unstable fixed point of the trajectory (the jacobian has one positive eigenvalue) as long as $g < \gamma$. Therefore, $X_f^T = (0,0,1)$ is a stable fixed point of the trajectory (the jacobian has only negative eigenvalues), whereas $X_i^T = (0,0,S_\infty)$ is an unstable fixed point. We can therefore solve the ODE in equation (S38) with the initial condition $X = X_i$ and let the trajectory converge toward X_f. Wave shapes for several values of γ are shown in Figure S5.

7 Level statistics and connectedness

7.1 The level spacing distribution of the flux matrix

We consider the level statistics of the symmetric matrix $F_{ab} = (f_{ab} + f_{ba})/(M_a M_b)$, where f_{ab} is the number of people residing in community a visiting community b during one day and M_a is the total population of site a, see equation (S2). The symmetric flux is related to the infectivity matrix through $\beta_{ab} = p\beta F_{ab} M_b$, where the factor $p\beta$ is estimated by a fit to the SafeGraph mobility data over $K = 353$ days from March 1st 2020 to February 16th 2021 and is given by $p\beta = 0.05 \pm 0.02$, as is shown in Figure 2**b** of the main text. Following common practices in the treatment of random matrices[38], the diagonal elements $F_{aa}^{(k)}$ of the K matrices of size $N \times N$ were drawn from a generic gamma distribution with mean $\mu_a^{(k)} = (N-1)^{-1} \sum_{b \neq a} F_{ab}^{(k)}$ and variance $\mu_a^{(k)}/2$ $\{k = 1, 2, \ldots, K\}$. To obtain the unfolded spectra[38], the eigenvalues E_{ak} are re-scaled as

$$\varepsilon_{ak} = N\bar{G}(E_{ak}), \qquad a = 1, 2, \ldots, N, \; k = 1, 2, \ldots, K \tag{S41}$$

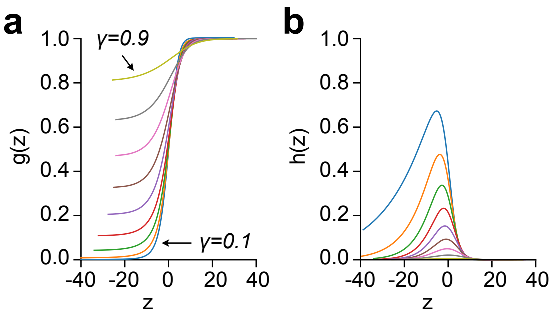

Figure S5. Shape of the SIR traveling waves: **(a)** $g(z)$ and **(b)** $h(z)$, obtained by solving equation (S38) for values of $\gamma = 0, 0.1, 0.2, 0.3, 0.4, 0.5, 0.6, 0.7, 0.8, 0.9$.

where $G(E)$ is the empirical level staircase function $G(E) \equiv (NK)^{-1} \sum_{ak} \Theta(E - E_{ak})$ (*i.e.* the cumulative eigenvalue probability function) and $\bar{G}$ is its smoothed interpolation[45–47]. The nearest level spacings $s_{ak} \equiv \varepsilon_{a+1,k} - \varepsilon_{a,k}$ are then normalized so that the average level spacing across the entire spectrum is equal to one for all values of k:

$$\bar{s}_{ak} = \sum_{a=1}^{N-1} s_{ak}/(N-1) = 1. \tag{S42}$$

Figure S6**a** shows the level spacing distribution of the unfolded spectrum, $p(s) \equiv N_s^{-1} \sum_{i=1}^{N_s} \delta(s - s_i)$, where N_s is the number of independent spacings (for K matrices, $N_s = K(N-1)$). The level statistics of the full matrix, Figure S6**a**, is significantly different than the level statistics obtained after all the links of distance larger than 170 km have been omitted (Figure S6**b**). Clearly, the empirical distribution $p(s)$ interpolates between the Wigner-Dyson distribution of the GOE ensemble (more precisely, the Wigner surmise[38]) that exhibits linear level repulsion typical of extended correlated states:

$$p(s) = (s\pi/2) e^{-s^2\pi/4} \qquad (s \geq 0), \tag{S43}$$

and the Poisson distribution describing independent localized states:

$$p(s) = \exp(-s) \quad (s \geq 0) \tag{S44}$$

In both equations (S43) and (S44), the average level spacing is normalized to unity: $\langle s \rangle = \int_0^\infty \mathrm{d}s\, s p(s) = 1$.

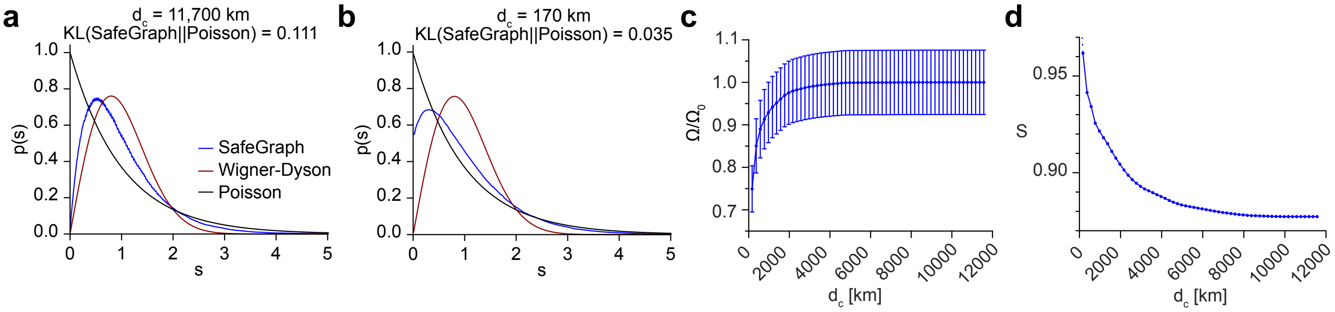

Figure S6. The unfolded level spacing $s_i = \varepsilon_{i+1} - \varepsilon_i$. **(a)** The level spacing distribution of the full matrix, *i.e.* without removing any links. **(b)** The level spacing of the truncated matrix $d_c \leq 170$ km. Here $\varepsilon_i = \bar{G}(E_i)$ $\{i = 1, 2, \ldots, N\}$, where $\bar{G}(E)$ is the smoothed energy staircase function. The GOE level spacing (equation (S43)) is plotted in red and the Poisson (equation (S44)) in black. **(c)** The relative epidemic size Ω/Ω_0 as a function of d_c. The estimated relative error is $\Delta\Omega/\Omega_0 = 7\,\%$. Ω_0 denotes the unrestricted epidemic size. **(d)** The entropy S(SafeGraph) as a function of the cutoff distance d_c in the range 170-12000 km. The maximal value of d_c corresponds to a full flux matrix.

7.2 KL divergence and the level spacing entropy

To quantify the difference between Figure S6**a** and Figure S6**b**, we have calculated the Kullback-Leibler divergence between the empirical spacing distribution, $p(s)$, and the Poisson distribution

$$D(\text{SafeGraph}||\text{Poisson}) = 1 + \sum_{i=1}^{N_s} p(s_i)\log[p(s_i)] = 1 - S(\text{SafeGraph}) \tag{S45}$$

where D is the KL divergence and S is the entropy of $p(s)$. The KL divergence of the full matrix is then $D \simeq 0.11$. The KL divergence of the truncated matrix, that shows significantly less level repulsion, and is closer to Poisson statistics, is smaller than that of the full matrix by a factor of three: $D \simeq 0.04$. The KL divergence between the GOE and the Poisson distributions is

$$\begin{aligned} D(\text{GOE}||\text{Poisson}) &= \frac{\pi}{2}\int_0^\infty ds s \exp(-s^2\pi/4)\left[\log(s\pi/2) - \pi s^2/4 + s\right], \\ &= (\log\pi - \gamma_E)/2, \\ &= 0.283057, \\ &\equiv D_0 \end{aligned} \tag{S46}$$

where $\gamma_E = 0.57721\ldots$ is the Euler constant. Therefore, in terms relative to D_0 we obtain: $D/D_0 = \{0.39, 0.12\}$ for the full and the truncated matrices respectively. Thus, $D(\text{SafeGraph}||\text{Poisson})$ is smaller than D_0 and approaches zero as links are being removed. Equivalently, since (i) the entropy of the Poisson distribution is equal to its average $\langle s\rangle = 1$ and (ii) the entropy of Wigner-Dyson distribution is $1 - D_0 \simeq 0.72$, the level spacing entropy varies in the range $1 - D_0 < S(\text{SafeGraph}) \leq 1$ and it approaches unity as links are removed and the states get localized.

The entropy as a function of the cutoff distance d_c is shown in Figure S6**d**. The distribution $p(s)$ is found by (i) removing all links of distance larger than d_c (ii) unfolding the spectra of the ensemble of K truncated matrices and (iii) repeating the procedure for each value of d_c. The corresponding size of the epidemic $\Omega(\infty)$ and its error $\Delta\Omega$ are estimated by solving equations (S12) and (S13) for each one of the truncated matrices and then calculating the mean and standard deviation over K samples. In the numerical computation we assumed a recovery rate $\gamma = 0.125\,\text{d}^{-1}$ and initial fraction of infections $\varepsilon = 10^{-4}$ uniform for all the communities. The epidemic size Ω/Ω_0 as a function of d_c, relative to the value of the unrestricted epidemic size Ω_0 (*i.e.* for the case of a full untruncated matrix), is shown in Figure S6**c**. Thus, a cutoff distance of $d_c \simeq 500\,\text{km}$ leads to a $10\,\%$ reduction in the epidemic size. Similarly, $d_c \simeq 200\,\text{km}$ reduces the epidemic size by $25\,\%$. These results are in accordance with the time evolution of the epidemic as is presented in Figure 3**b** of the main text.

7.3 Connectedness and epidemic size

The crossover from Wigner-Dyson to Poisson distribution as links between communities are being successively removed, suggests a policy of isolation which can lead to an effective reduction of the epidemic size. Edges connecting vertices a and b can be removed in several ways. For example, (i) according to the geographical distance $d(a,b)$ as has already been done in section 7.2, (ii) according to the "nominal" distance $|a-b|$, or (iii) by using the edge-betweenness centrality index, as proposed by Girvan and Newman[42]. Note, that in each one of these cases, one can as well consider a moderated mitigation policy: instead of eliminating links completely, one can impose constraints on the flux of people that are allowed to commute via central pre-determined links.

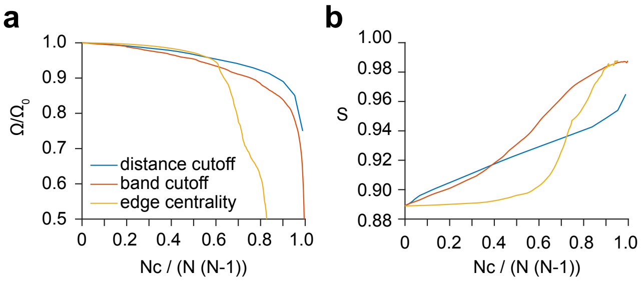

Figure S7. **(a)** The epidemic size Ω/Ω_0 versus the relative number of cuts $N_c/N/(N-1)$ for the 3 mitigation methods: distance cutoff, bandwidth, and edge-betweenness centrality. Ω_0 is the epidemic size for $N_c = 0$. **(b)** A comparison of the entropy versus the relative number of cuts for the three mitigation method.

To study mitigation according to the nominal distance $|a-b|$, we consider the ensemble of banded matrices F_{ab} with half-bandwidth $B \equiv \max_{F_{ab}\neq 0}|a-b|$, such that the number of non-zero diagonals is $2B+1$ irrespective of the underlying

physical distance. The increase of Ω/Ω_0 as a function of B, along with a corresponding decrease of the entropy $S(\text{SafeGraph})$, is shown in Figure 4**d** of the main text. Comparing to Figure 4**c** of the main text, a reduction of 10 % in Ω/Ω_0 amounts to entropy values of $S = \{0.94, 0.98\}$ for the geographical and nominal cutoffs, respectively. One may argue that mitigation based on "nominal" distance is meaningless, because such a distance changes under the permutations of sites. Indeed, a certain link may either be kept as is, or be omitted, according to an arbitrary re-ordering of communities. However, performing such permutation only reshuffles the matrices of the random ensemble among themselves. Consequently, for $K, N \gg 1$, the level statistics and the resulting epidemic size are hardly affected.

Centrality indices have been used for detecting the modular structure of social and biological networks[42,43]. The edge-betweenness centrality of edge (a,b) is defined as[44]: $C(a,b) = \sum_{ts} \sigma_{ts}(a,b)/\sigma_{ts}$, where (t,s) stands for "target" and "source" vertices, respectively. Here, σ_{ts} are shortest paths going $t \leftarrow s$ and $\sigma_{ts}(a,b)$ are such paths which, in addition, go along edge (a,b). The entries σ_{ts} are weighted by the flux that they can carry (otherwise, unweighted paths take only binary values $\sigma_{ts} = 0, 1$). The summation is carried over all (t,s) pairs that are different from (a,b). As observed in[42], edges running between loosely-connected communities should have high values of C.

The scheme proposed in[42] for identifying communities in a network is as follows: (i) calculate C for all edges of the network (ii) remove the edge with highest C (iii) re-calculate C for all edges affected by the removal. (iv) Repeat from step (ii) until no edges remain. Here, we are using the same procedure for mitigating the epidemic. There are few practical modifications though:

- Edge-betweenness is calculated for the mean matrix $H_{ab} = \sum_k F_{ab}^{(k)}/K$. The removal of links is then applied to each matrix separately.

- For a faster computation of step (ii), edges are removed in chunks rather than one-by-one. This is done with the help of the efficient shareware package MatlabBGL that is able to rank all the edges of H in a single instance. The initial size of a chunk is 10000 (about 1 % of the total number of links in H) and it then decreases adaptively.

- The computation terminates when the resulting KL divergence is sufficiently close to zero. Our modified procedure is clearly sub-optimal. We found, however, that compared to the exhaustive search (i)-(iii), the results almost don't change because the variations of the KL divergence at the initial stages are relatively small.

The entropy $S(\text{SafeGraph})$ and the size of epidemic Ω/Ω_0 as a function of the number of removed links, N_c (*i.e.* number of cuts), are shown in Figure S7. The size of epidemic drops abruptly when the relative number of cuts $c \equiv N_c/N/(N-1)$ exceeds the value $c = 0.6$ (from a relative size of 95 % at $c = 0.6$ to 75 % at $c = 0.7$). This drop is accompanied by the increase of entropy around the same value of c. Thus, mitigation based on edge-betweenness performs better than either the distance or the nominal cutoffs, none of which exhibits a similar abrupt crossover. The betweenness index outperforms the other two methods mainly because it is sensitive to the flux that is flowing through links. The "transition" at $c = 0.6$ is expected to become much sharper as $N \gg 1$ approaches the thermodynamic limit. The evolution of $p(s)$ as links are gradually being removed is demonstrated in movies S5-S7.
